# Reasons for encounters and psychiatric comorbidity in an urban Bavarian primary care out-of-hour service - results of a cross sectional study

**DOI:** 10.1186/s12913-017-2749-3

**Published:** 2017-11-28

**Authors:** Constanze Storr, Lucia Marieke Gahbler, Klaus Linde, Antonius Schneider

**Affiliations:** Institute of General Practice, University Hospital Klinikum rechts der Isar, Technische Universität München, Orleansstr. 47, 81667 Munich, Germany

**Keywords:** Out-of-hour primary care, Psychiatric comorbidity, Reason for encounter, General practitioner office, Utilization rate, Sociodemographic variables

## Abstract

**Background:**

International studies have shown a contribution of psychiatric comorbidity to high utilization rates in out-of-hour primary care (OOHC). Up to now, the impact of psychiatric comorbidity in German OOHC remains unclear. Therefore, we aimed to investigate reasons for encounter (RFE), possible psychiatric comorbidity, utilization rates, and a possible association between utilization rate and psychiatric comorbidity among patients of an urban OOHC unit.

**Methods:**

In a cross-sectional, prospective, naturalistic study five hundred self-referred patients completed a self-designed questionnaire addressing RFE, past office visits and personal information. Additionally, we employed three validated questionnaires (PHQ-9, PHQ-15 and GAD-7) to screen for mental disorders. We collected information about past visits through computerized patients’ charts. Diagnoses were classified according to the International Classification of Primary Care-2.

**Results:**

The most frequent RFE were musculoskeletal complaints (36%), followed by respiratory diseases (13%), gastrointestinal problems (10%), skin conditions (8%) and urologic ailments (6%). Of the included patients 58% were working fulltime and 61% had greater than or equal to 10 years of education. The mean age was 37.3 in females and 40.5 years in males. Prevalence of psychiatric comorbidity was 27%. Only 3% visited the office more than twice over a 12 months period. We could not find an association between high utilization and psychiatric comorbidity.

**Conclusion:**

In this study, musculoskeletal complaints were the most frequent RFE. Patients were predominantly young, employed and educated. The prevalence of psychiatric comorbidity was similar to the prevalence in common general practitioner offices and showed no significant relation to frequent attendance. This information might help to prepare physicians better for patient care in OOHC.

## Background

European countries have implemented out-of-hour care (OOHC) in their systems over the last 20 years, but there is only little knowledge about the German urban out-of-hour primary care [1]. OOHC in Bavaria, Germany, is delivered through general practitioners (GPs) without fixed appointments to patients with acute medical problems at times when the GP offices are closed. In rural Bavaria OOHC mainly takes place in GP offices. In urban Bavaria OOHC is mostly (87%) located on the premises of hospitals [2]. In 1998, an OOHC was established at the university hospital of the Technical University of Munich (TUM). The OOHC is run by self-employed general practitioners like most OOHC in Bavaria. There is a close cooperation (e.g. referrals) between the clinic and the hospital for patients seeking immediate help. An important objective is relieving the emergency department (ED) from high patient volume on the weekends and late hours, despite available international evidence does not convincingly show OOHC actually to do so [3]. On the other hand, some centers report misuse or overuse including an increase in non-injury related problems and an increase of older patients using OOHC [4]. There is evidence that most ED patients belong to OOHC [5]. In the same study walk in distributions (self-referrals without appointments) were equal in both locations, but entire outpatient treatment was more likely to occur in OOHC than in ED [4, 5]. Following this observation, OOHC have been established and developed in different regions of Bavaria, Germany.

Nevertheless, there is a lack of information on what kinds of patients actually seek medical attention in urban OOHC in Germany. Studies from several European countries found 12-month prevalence rates of psychiatric comorbidity amongst adults visiting GP offices ranging from 9% to 27% [6]. A Dutch study showed that psychiatric comorbidity has high impact on utilization rates in OOHC [7]. Up to now, there are no German studies investigating the impact of psychiatric comorbidity on OOHC utilization. We investigated both the reasons for encounter (RFE) and screened for common mental disorders (anxiety disorders, affective disorders and somatoform disorders) in an urban OOHC [8]. In addition we collected the number of patients frequently using the OOHC and investigated a possible association of utilization rate and psychiatric comorbidity.

## Methods

We performed a cross sectional prospective naturalistic study in an OOHC located on the premises of university hospital of the Technical University of Munich. The study was approved by the local ethics committee and carried out from April to July 2012. Due to administrative reasons, the in-depth analysis was finally performed in 2016. The OOHC was open on weekdays from 7 to 10 pm, on Wednesdays and Fridays from 4 to 10 pm, on weekends and bank holidays from 9 am to 10 pm. Patients were eligible if they were older than 18 years, had sufficient knowledge of German language, adequate mental performance and signed an informed consent. One medical student (LMG) offered all consecutive patients in the waiting area of the OOHC to participate in the study. In case of refusal, we gathered information about gender and age for non-participant analysis. After signing the informed consent, all participants were asked to fill a seven-page questionnaire, which included information on the actual RFE, on the number the patient had been in treatment for these complaints previously and on sociodemographic characteristics. Subsequently we classified the patient provided RFE according to the International Classification of Primary Care-2. The questionnaire also included a German version of the Patient Health Questionnaire (PHQ-D) [9], and the Generalized Anxiety Disorder Assessment (GAD-7) [8] for generalized anxiety screening. The PHQ-D consists of three modules. The PHQ depression section (PHQ-9) comprises nine items, which score each of the DSM-IV criteria (Diagnostic and Statistical Manual of Mental Disorders). The PHQ-15 consists of 15 items to screen for somatization and to monitor somatic symptom severity. The panic disorder section includes 15 dichotomous questions for a diagnosis based on DSM-IV-criteria. Following the physician’s consultation, information on diagnosis (we classified the diagnosis according to the full ICPC-2), initiated therapy and previous visits over the last 12 months were collected.

Data were analyzed using SPSS Statistics 19 and 23 software (IBM, New York, USA). Depending on the scale level data were summarized descriptively using absolute and relative frequency, median and range or mean and standard deviation. Currently there is no consistent definition of ‘frequent attender’ in GP offices. Given the low numbers of frequent attenders in our data set we implemented at least two OOHC visits over the last 12 months as cut-off for use in our statistical analyses. We performed an additional sensitivity analysis using at least three visits as a cutoff. Fisher’s exact test was used to investigate whether prevalence of psychiatric morbidity (minor/major depression, somatoform disorder, generalized anxiety disorder, any of these) was different among frequent attenders. Subgroup analyses of sociodemographic characteristics according to gender were done using Fisher’s exact-, Chi^2^- and Student’s t-test. To investigate whether sex, age, school education (at least 10 years or less), marital status and employment status were associated with at least one mental comorbidity (according to the screening instruments used) or being a frequent attender we performed multivariate logistic regression analyses. We aimed for a target of 500 patients to meet a precision (95% confidence interval (95%CI)) of 1.2% assuming a prevalence of frequent attenders at 2%.

## Results

### Recruitment

On the 38 recruitment days from April to July 2012 we contacted a total of 888 persons (mean 23 per day) in the OOHC. Of these 122 were not eligible (82 had insufficient understanding of German language, 39 were younger than 18 years). Among the 767 eligible patients, 267 refused to participate. Patients ineligible or refusing were more frequently female than participants (62.9% vs. 54.8%; *p* = 0.02) and trended to be younger (40.5 years vs. 38.8 years; *p* = 0.23). A total of 500 patients (65.2% of all approached) signed consent and participated. Female patients (*n* = 274) were significantly younger over male patients (mean 37.3 vs. 40.5 years; *p* < 0.001; see Table 1). There were no significant differences between male and female patients in terms of educational or marital status. Two hundred ninety one (58.2%) of the patients were working fulltime, with significantly more men than women working fulltime (*p* < 0.001).

**Table 1:** Sociodemographic characteristics of participants. Values are means (standard deviations) or absolute frequencies (percentages)

	Women (*n* = 274)	Men (*n* = 226)	Total (*n* = 500)	*p*-value
Age	37.3 (14.9)	40.5 (14.4)	38.8 (14.8)	0.001
≥ 10 years education	222	179	401 (83.9%)	0.199
Employment				0.001
- fulltime	128	163	291 (61.0%)	
- part time	51	17	68 (14.3%)	
- homemaker	13	2	15 (3.1%)	
- pensioner	24	22	46 (9.6%)	
- unemployed	6	3	9 (1.9%)	
Marital status				0.344
- married	170	157	327 (68.0%)	
- single	84	58	142 (29.5%)	
- widowed	7	5	12 (2.5%)	

*P*-values from Student’s t-Test, Fisher’s exact test or Chi^2^-test

### Reasons for encounters

The physicians recorded RFE in 487 patients (97.4%; see Table 2). In 13 patients, diagnoses were lacking. Up to three RFE could be specified. In 412 (82.4%) patients, the physician recorded one RFE. In 68 (13.6%) patients, he/she specified two reasons and seven (1.4%) patients had three RFE. Therefore, we recorded 562 different RFE, which we coded by ICPC-2 chapters. The exact breakdown with full ICPC-2 of the diagnoses made by the GPs showed that lower back pain accounted for 5.7% (32 patients, which was the most frequent RFE, followed by urinary tract infections (5.2%), post coital contraception (4.6%) and insect bites (3.9%; Table 3). Therefore, eleven RFE account for more than one third (36.1%; Table 3).

**Table 2:** Reasons for encounters (physicians recorded) with ICPC-2 coding in 487 patients

Reasons for encounters ICPC-2 chapters	Absolute frequency (percentage)
Musculoskeletal L	200 (35.6%)
Digestive system D	57 (10.1%)
Respiratory system R	73 (13.0%)
Skin S	45 (8.0%)
Neurological N	28 (5.0%)
General A	30 (5.3%)
Urological U	31 (5.5%)
Pregnancy, birth family planning W	28 (5.0%)
Cardiovascular system K	28 (5.0%)
ENT H	10 (1.8%)
Procedures C	3 (0.5%)
Blood, blood building organs, immune system B	9 (1.6%)
Psyche P	7 (1.2%)
Ophthalmology F	2 (0.4%)
Endocrine, metabolic, nutrition T	7 (1.2%)
Female genitals X	2 (0.4%)
Male genitals Y	2 (0.4%)
Social issues Z	0 (0%)
Total number of RFMC	562 (100%)

**Table 3:** Most frequent reasons for encounters in detail (physician recorded), eleven reasons for medical consultation account for more than one third (36.1%)

Reasons for encounters according to full ICPC-2	Absolute frequency (percentage)
Lower back pain (L03)	32 (5.7%)
UTI (U71)	29 (5.2%)
Post coital birth control (W10)	26 (4.6%)
Insect bites (S12)	22 (3.9%)
Ankle sprain (L16)	16 (2.9%)
Upper respiratory infection, acute (R74)	15 (2.7%)
Acute bronchitis/bronchiolitis (R78)	14 (2.5%)
Back pain (L86)	13 (2.3%)
Upper back pain (L01)	12 (2.1%)
Chest pain (K01)	12 (2.1%)
Tonsillitis, acute (R76)	12 (2.1%)
Total number	203 (36.1%)

### Frequency of OOHC visits

Four hundred thirty seven patients (87.4%) visited the office only once (on the present day), 44 (8.8%) twice, and 15 (3.0%) three to six times during a 12 months period (based on computerized charts). The question whether patients already had consulted their general practitioner in the past for the same medical condition, was answered positive by 144 patients (29.5%), 22 did not make further specifications. Of all respondents, 122 patients consulted their primary care physician within a range of up to 30 visits over the last 12 months. Nineteen of the 500 patients (3.8%) got a sick leave form. The minimum period of absence was 1 day; the maximum was 4 days (mean 2.2 days, median 2 days).

### Psychiatric comorbidity and high utilization

According to the used screening instruments 148 (29.6%) patients had at least one suspected psychiatric comorbidity; 97 (18.4%) of the patients had only one mental condition, 31 (6.2%) had two, 15 (3.0%) three and five patients (1.0%) had four mental conditions. There was no association between psychiatric comorbidity and high utilization (Table 4). Of the included patients 129 (29.5%) who had visited the OOHC only once over a 12-months period had at least one psychiatric comorbidity compared to 18 (30.5%) with two or more visits. Among the 15 patients with three or more visits 5 (33.3%) had at least one psychiatric comorbidity.

**Table 4:** Psychiatric comorbidities tested by PHQ-D and GAD-7 in all participants and in patients with ≥ 2 vs. only one visit to the PCOOHS over the last 12 months

Diagnosis (n missing across all participants/in subgroup analysis)	All participants (*n* = 500)	Subgroup analysis		Odds ratio (95% CI)
		≥ 2 visits (*n* = 59)	Only 1 visit (*n* = 437)	
Minor or major depression (15/17)	92 (18.4%)	12 (21.1%)	80 (18.9%)	1.15 (0.58; 2.27)
Somatoform disorder (17/21)	69 (13.8%)	5 (9.1%)	64 (15.1%)	0.56 (0.22; 1.47)
Moderate or severe anxiety (18/22)	37 (9.4%)	6 (11.1%)	41 (9.7%)	1.17 (0.47; 2.89)
Panic disorder (5/9)	16 (3.2%)	2 (3.4%)	13 (3.0%)	1.13 (0.25; 5.14)
At least one mental comorbidity (2/4)	148 (29.6%)	18 (30.5%)	129 (29.5%)	1.05 (0.58; 1,89)

### Logistic regression analysis

In logistic regression analyses, we did not find any association between sex, age, school education, marital status, and being employed with a suspected mental disorder or having attended the OOHC at least twice.

## Discussion

### Main findings

In this study, musculoskeletal complaints were the most frequent RFE. Patients were predominantly young, employed and well educated. The prevalence of psychiatric comorbidity was similar to that in a common GP office and the number of frequent attenders was low. We could not find an association between utilization more than twice and psychiatric comorbidity.

### Strengths and limitations

Our study provides information on how patients utilize an urban OOHC in Bavaria, Germany. To our knowledge, it is the first study to investigate a possible link between psychiatric comorbidity and high utilization in OOHC. The setting of the researched OOHC is comparable to other hospital based OOHC in Bavaria, Germany [2].

We included 500 patients in our cross-sectional, prospective naturalistic study; thus, our sample size is small, compared to larger international studies. Another limitation of our study is that participants might not be fully representative for all patients seeking care at the unit. Since recruitment occurred from April to July a seasonal bias might be possible and a longer recruitment period may have resulted in a different ranking of RFE. Since we aimed to investigate psychiatric comorbidity we had to use appropriate instruments to screen for mental disorders. Therefore, only adult patients with sufficient knowledge of German language were eligible. One third of the eligible patients refused participation. This may be due to their conditions we found in our study (patients did not want to be bothered with an extensive questionnaire) and the anonymity of the OOHC compared to their usual general practitioner. Furthermore, due to logistical reasons, recruitment was not possible on all days during the observation period rather than largely on weekends and bank holidays. Frequent attenders may be less common on these days compared to evening hours on working days due to the known long waiting times. It should also be noted that the opening hours of the OOHC studies do not cover nighttime.

The lack of an association of utilization and mental comorbidity should be interpreted with caution. Since only 15 patients had visited the OOHC more than twice we performed regression analysis comparing participants attending, once, twice or more frequently. It should also be kept in mind that the prevalence of mental disorders was determined using screening questionnaires and not employing a structured clinical interview (the reference standard for diagnosis) causing a potential risk of misclassification. However, since numerous health service research studies and epidemiological studies use the PHQ and the GAD-7 our findings compare well with other studies employing these tools.

### Interpretation

The RFE in our population are similar to those found in two Dutch studies [7, 10]. In contrast RFE differ to some extent from those found by Leutgeb et al. in an analysis of electronic charts of a strictly consecutive sample of 15,886 patient contacts in one rural OOHC in Germany over a 3 years period [11]. The most frequent RFE in this population were fever (5.8%), sore throat (4.8%) and cough (4.8%). Lower back pain accounted for 3.9%. Differences may partly be due to the selection of the patients in our study (see above). The different setting (rural vs. urban) is likely to play a major role in the observed differences. For example, we excluded children and adolescents, resulting in a higher mean age compared to the rural OOHC population (39 vs. 42 years). Furthermore, the share of female patients was higher (65.9% vs. 54.8%). The distribution of males (45.2%) and females (54.8%) in our study compares well with results of other studies in German GP offices [12, 13]. Compared to results of a study conducted in common GP offices in Bavaria, Germany, there are clear differences [6]. While musculoskeletal complaints are still the most frequent ICPC-2 category they are less prevalent (19%) compared to our study. The following categories are procedures (i.e. vaccinations, checkups (14%), respiratory diseases (12%), cardiovascular diseases (12%) and psychological conditions (11%). The lower frequency of mental disorders in OOHC may be partly due to the focus on acute problems and the lack of physician’s familiarity with the patient. Vice versa, ICPC-2 chapters frequent in the OOHC are less frequent in the GP office (digestive complaints 10.1% vs. 8%; urologic issues 5.5% vs. 2%) [6]. Another study using data of EurOOHnet (European research network for out-of-hour primary health care) compared RFE in eight European countries in spring 2009. This study found that respiratory conditions were most frequent (20.4%) followed by musculoskeletal problems (15%). Even though the percentages differ in the ranking of the top five ICPC chapters, our findings are largely similar: musculoskeletal, digestive, respiratory, general and unspecified, as well as skin represent the top five ICPC chapters [14]. A Belgian study from 2010 compared RFE in general practice and emergency departments. While ‘respiratory’ (36.8%) and ‘digestive’ (20.2%) were most frequent at GP services, ‘musculoskeletal’ (21.6%) and ‘skin’ (17.3%) were the most frequent chapters in ED [15]. Our study confirms another investigation in Germany which showed lower back pain being the most frequent diagnosis in OOHC [16].

According to the screening instruments used, 27.2% of our participants met criteria of at least one psychiatric comorbidity during the last 4 weeks. In our study 18.4% of patients screened positively for minor or major depression, followed by somatoform disorders (13.8%) and moderate or severe anxiety (9.4%). This prevalence is well in agreement to findings in samples of the general population in Germany and other European countries [17–19]. In our previous study conducted in common GP office, the point prevalence was 26.2% [6]. This suggests that the prevalence of mental disorders as measured by screening tools is similar across the different settings. However, general practitioners actually record the clinical diagnosis of a mental disorder less frequently during regular office hours (10.7%) and also particularly in OOHC [6]. Little knowledge about the patients’ history in the OOHC setting and the lack of time may contribute to this. OOHC physicians should consider the high psychiatric comorbidity to grant optimal care. Timely identification of ʻdifficultʼ patients may help to prevent somatic fixation as well as over-treatment. However, we found no significant relation of frequent attendance and psychiatric comorbidity.

Only 3.0% visited the OOHC more than twice over the last 12 months. These patients were responsible for 9.1% of all visits during the study period. This is less than observed in other studies [7, 12, 20]. Possible reasons for this are well-known long waiting times, visits on weekends and the option to terminate participation in our study. On the other hand, we found that 144 (29.5%) participants (only 122 made further specifications) already had consulted their own general practitioner for the same reason with up to 30 visits in the past (mean = 3, median = 1.5, SD = 4.6). Even though we only found 3% frequent attenders in the investigated OOHC, it may be possible that some of the other included patients are frequent attenders in their own GP office. Concerning the sociodemographic values our study is well in line with other studies reporting that younger patients and fulltime workers more frequently use an OOHC [16].

## Conclusion

In a German urban OOHC the most frequent RFE are related to musculoskeletal complaints. In addition, diseases of digestive and respiratory systems also showed high prevalences. Patients in the German urban OOHC were predominantly young, employed and educated. The prevalence of psychiatric comorbidities was similar to prevalences reported from common general practitioner offices and showed no significant relation to frequent attendance. These findings may help to prepare physicians better for patient care in OOHC.

## Abbreviations

DSM-IV: Diagnostic and Statistical Manual of Mental Disorders IV
ED: Emergency Department
EurOOHnet: European research network for out-of-hour primary health care
GAD-7: Generalized Anxiety Disorder Assessment
GP: General practitioner
ICPC-2: International Classification of Primary Care-2
OOHC: Out-of-hour primary care
PHQ-15: Patient Health Questionnaire-15
PHQ-9: Patient Health Questionnaire-9
RFE: Reasons for encounter
TUM: Technical University of Munich
